# Trends in formal care by age and time to death: the use of healthcare and care-home facilities in Finland between 2005 and 2018

**DOI:** 10.1093/eurpub/ckaf061

**Published:** 2025-04-27

**Authors:** Luca Dei Bardi, Margherita Moretti, Laura Cacciani, Kaarina Korhonen, Pekka Martikainen

**Affiliations:** Helsinki Institute for Demography and Population Health, University of Helsinki, Helsinki, Finland; Max Planck—University of Helsinki Center for Social Inequalities in Population Health, Helsinki, Finland; Helsinki Institute for Demography and Population Health, University of Helsinki, Helsinki, Finland; Max Planck—University of Helsinki Center for Social Inequalities in Population Health, Helsinki, Finland; Department of Epidemiology - Regional Health Service, ASL Roma 1, Rome, Italy; Helsinki Institute for Demography and Population Health, University of Helsinki, Helsinki, Finland; Max Planck—University of Helsinki Center for Social Inequalities in Population Health, Helsinki, Finland; Helsinki Institute for Demography and Population Health, University of Helsinki, Helsinki, Finland; Max Planck—University of Helsinki Center for Social Inequalities in Population Health, Helsinki, Finland; Max Planck Institute for Demographic Research, Rostock, Germany

## Abstract

Population ageing may further increase formal care use. Strong predictors in addition to age include time to death and the cause of death. The aim of this study is to analyse trends in the use of formal care in Finland by these factors. We analysed patterns of care use among all Finnish residents who died at the age of 65 and older between 2005 and 2018 (263 660 men and 315 439 women), linking different administrative registers. We used multinomial logistic models to examine the time spent in healthcare and care-home facilities, stratifying our analyses by gender and time to death. Between 2005 and 2018, formal care use became increasingly concentrated in the last years of life for all causes of death, and the effect of age slightly diminished. However, in 2017–18, decedents aged 65 in their last year of life spent up to seven months less in care than their counterparts aged 105. Over time, unadjusted per-capita care usage in the last seven years of life increased from 9.3 to 10.5 months for men, and from 16.8 to 19.3 months for women. Concurrently, the total time spent in care on the population level increased by 44%. Age and time to death are major determinants of formal care use. An increasing age at death drove the increase in per-capita and total care usage over time. Population ageing will continue to increase future care needs; governments must prepare for this scenario.

## Introduction 

Population ageing is driven by ongoing demographic shifts, in particular notable increases in life expectancy [1, 2] and past fertility fluctuations. Nevertheless, increases in life expectancy do not inherently equate to improved population health, and a longer lifespan is associated with many indicators of poor health [3]. Older age is also strongly related to the prevalence of multimorbidity, dementia, and other forms of cognitive impairment, which in turn increases the need for service utilization [4, 5]. Previous studies have reported that individuals dying at older ages have ‘slower deaths’, experiencing dependency and impairment more frequently than younger people regardless of the cause of death [6]. The future sustainability of public care following a potential increase in demand for old-age care services has also been discussed [7, 8].

The extent to which a potential increase in care use could be associated with population ageing was questioned in research conducted by Zweifel and colleagues [9]. They found that the primary determinant of hospital-care utilization was the Time To Death (TTD) rather than the chronological age of the individual, arguing that people in their last years of life consume more care resources than those who survive to the same age. The focus of research and policy on chronological age has been defined as a ‘red herring’ (something misleading or distracting), diverting the debate away from improving the efficiency of the care system [9]. Following the red herring hypothesis, the relationship between age and care use would merely stem from the fact that older individuals are closer to death than younger individuals. In an ageing population, therefore, care use would be postponed to older ages without an increase in usage among individuals over their life course. Previous studies exploring the relationship between ageing and care expenditure have reported mixed conclusions, partly due to the varying empirical strategies and national differences in the sociodemographic contexts [10]. While the red herring hypothesis may apply to acute care, evidence suggests that age is more relevant than TTD for determining the use of non-acute care [11].

Finland, in line with other high-income countries, has experienced rapid population ageing in recent decades. Over the past few years the number of people aged 65 and over increased by 363 672, from 16% of the total population in 2005 to 22% in 2018 [12]. In this context, Finland is currently one of the oldest countries in Europe, and the oldest among the Nordic states [13], with those aged over 65 projected to reach 27% of the population by 2035 [14]. It thus offers a unique demographic context in which to study care use at the end of life. Furthermore, the exhaustive register data covering the entire population allows research to be carried out free of self-report bias and loss to follow-up. Previous studies on Finland have confirmed that age has been a more important factor affecting care use than TTD, especially in nursing homes [15, 16]. Studies focusing on stays in care facilities of over 90 days (long-term care) during the last years of life have documented the marginal relevance of TTD compared to age at death (age), and have highlighted the care burden of dementia [17–20]. Although long-term care has become more concentrated at the end of life in recent decades [19–22], its total use has increased because of the growing number of older individuals with dementia [20]. Concentration of long-term care at the end of life could be partly attributed to policies that have restricted eligibility criteria and targeted care increasingly to those with more severe disabilities [23]. Over time, traditional institutions of long-term care, such as nursing homes, have largely been replaced with residential care facilities offering 24-h assistance. This was allegedly to facilitate ageing in a home-like environment [24], but the transfer of costs from municipalities to customers has certainly played a role. In fact, municipalities fully cover the costs of traditional nursing homes, whereas customers residing in residential care facilities with 24-h assistance pay for rent and services themselves.

The aim of this study was to provide a comprehensive and up-to-date analysis of the evolution and drivers of care use in the last seven years of life. In particular, our goal is to analyse formal care usage trends in Finland between 2005 and 2018 by age and TTD, while considering different types of care and causes of death.

## Methods

Drawing from Finnish register data, we gathered information on all residents in Finland who died between 2005 and 2018 at age 65 and older. We obtained information on gender, completed age at death, date of death, and the cause of death from Statistics Finland’s population and death registers. Causes of death were classified according to the International Classification of Diseases 10th Revision (ICD-10), grouped into the following six categories: malignant neoplasms (C00–C97), dementia and Alzheimer’s diseases (F01, F03, G30, R54), ischaemic heart diseases (I20–I25), cerebrovascular diseases (I60–I69), diseases of the respiratory system (J00–J64, J66–J99), and all other causes. Supplementary Fig. S1 shows the cause-of-death distribution across the study years.

The outcome variable was the number of days spent receiving formal care in healthcare or care-home facilities on the 2555 days before death (seven 365-day years), collected from the registers of the Finnish Institute for Health and Welfare. We considered facilities for healthcare and care homes separately. In line with the grouping used in previous studies [15, 16], healthcare refers to stays in hospitals and health centres, whereas care-home facilities include nursing homes, service homes with 24-h assistance, and rehabilitation centres [24, 25] (see Supplementary Material for additional details). If an individual was registered in more than one facility on the same day (e.g. hospital visit during a nursing-home stay), priority was given to healthcare. Individuals were classified as living at home if they were not present in either healthcare or care-home facilities. Data on formal home-based care services were not available and thus could not be assessed.

For descriptive purposes, we calculated the average number of months of formal care stratified by combinations of gender, 10-year age group, year of death, cause of death and TTD, and also by type of care for the supplementary analyses. We then ran age-year multinomial logistic Vector Generalized Linear Models [26] (VGLM), and finally our complete models, including causes of death. All models were stratified by gender and TTD, and included all possible interactions (see Supplementary Material for more details). The model results are shown as predicted months (days ÷ 30.41667) spent in care (i.e. time not at home) and in healthcare and care-home facilities separately (Supplementary Material). The Statistics Finland Board of Ethics and the Social and Health Data Permit Authority Findata approved the study (permit numbers TK/3343/07.03.00/2023 and THL/499/14.06.00/2024), and the data were pseudonymized before being provided to the researchers. All the analyses were performed using the VGAM package and R software [26, 27].

## Results

A total of 263 660 men and 315 439 women aged 65+ died in Finland between 2005 and 2018. The number of deaths increased from 16 156 for men and 20 323 for women in 2005 to 21 878 (+35%) and 24 743 (+22%) in 2018, respectively (Table 1). The age groups driving these increases were men aged 80 or older (+4470) and women aged 90+ (+3396). Concurrently, the average time spent in care in the last seven years of life increased from 13.5 to 15.2 months (+13%), the highest absolute and relative increases being in the 80–89 age groups for both genders. In line with the increases in the number of deaths and in the per-capita use of care, the months of care provided by the formal care system increased from 492 000 to 708 000 (+44%).

**Table 1.** Average number of months spent in care facilities during the last seven years of life (avg. months) and number of decedents (*N*), by gender, age at death, and year of death^a^

		2005		2018		Change (%)	
Gender	Age at death	Avg. months	*N*	Avg. months	*N*	Avg. months	*N*
All	All	13.5	36 479	15.2	46 621	13	28
	65–69	5.0	3397	5.3	4313	6	27
	70–79	8.5	11 360	8.8	12 113	4	7
	80–89	15.1	15 174	16.5	18 682	9	23
	90+	22.8	6548	23.5	11 513	3	76
Men	All	9.3	16 156	10.5	21 878	12	35
	65–69	4.5	2289	4.9	2794	9	22
	70–79	7.3	6579	7.5	7326	3	11
	80–89	11.5	5682	12.8	8583	11	51
	90+	17.0	1606	16.1	3175	−5	98
Women	All	16.8	20 323	19.3	24 743	15	22
	65–69	6.0	1108	6.0	1519	1	37
	70–79	10.1	4781	10.8	4787	6	0
	80–89	17.3	9492	19.7	10 099	14	6
	90+	24.7	4942	26.3	8338	7	69

a Decedents aged 65 or older in Finland in 2005 and 2018.

**Figure 1.**
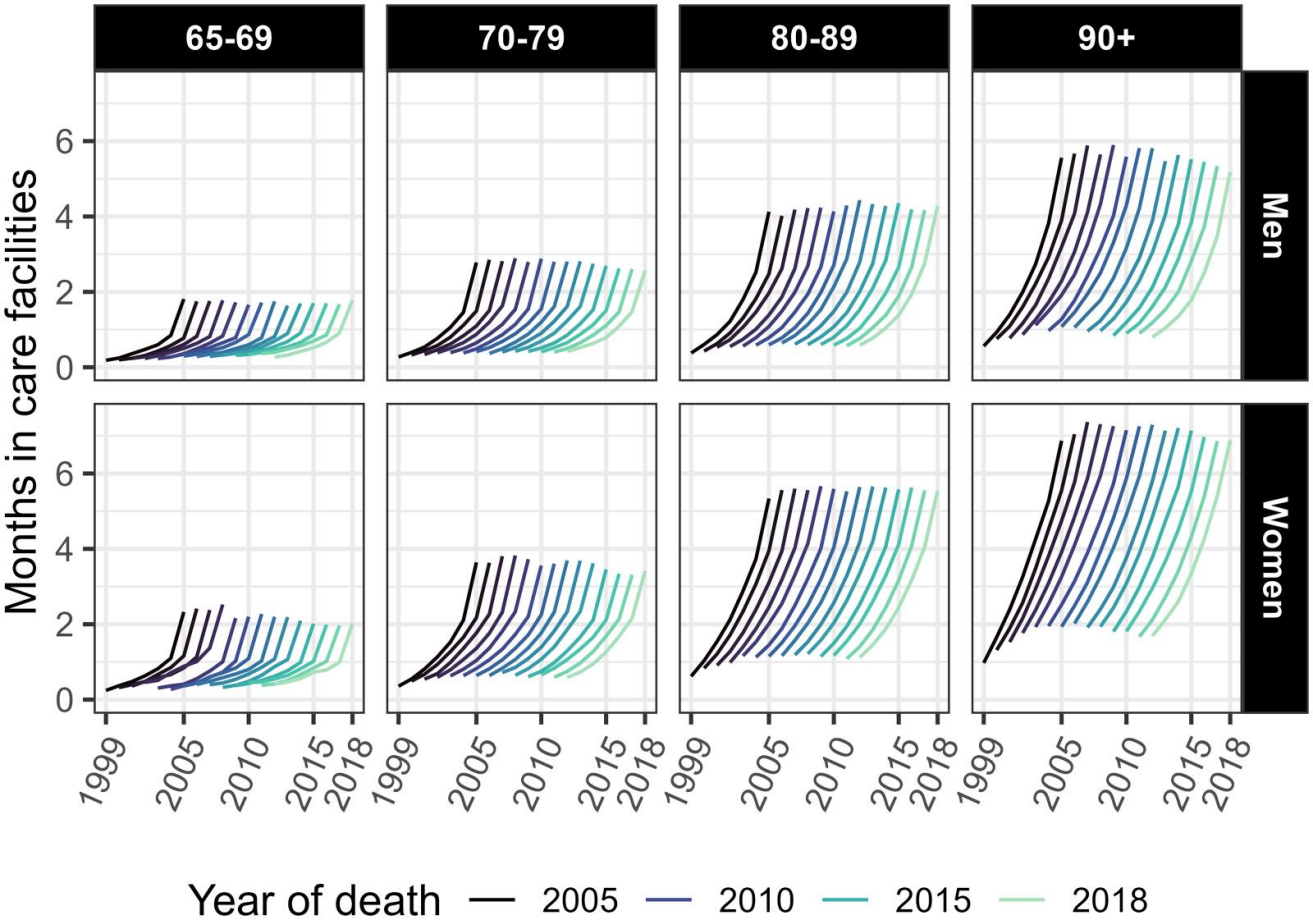
Average number of months spent in care facilities in the last 7 years of life by age at death, gender, year of death, and time to death. Decedents aged 65 or older in Finland between 2005 and 2018.

As Fig. 1 shows, formal care use over time remained quite stable between 2005 and 2018. On average, people spent between zero and two months in care seven years before death in all age groups and years. In contrast, time spent in care during the last year of life varied from two months for people aged 65–69 to seven months among women aged 90+. As death approached, the use of formal care increased more gradually and linearly in the older age groups, and sharply only during the very last years of life among younger decedents. The level of usage was strongly associated with age for both genders, the youngest decedents having about two months of total care in their last year of life, similar to that of the oldest decedents six to five years before death. Supplementary Fig. S2 shows the trends in the use of formal care over time broken down by the type of care. The time spent in healthcare facilities by TTD followed a J-shaped curve, following slower increases during the years further from death and an acceleration in the last years of life, which roughly accounted for half of the seven-year usage among younger decedents. Conversely, the increase in care-home use followed a more gradual and linear trend, although it also moved towards J-shaped curves in more recent years. A comparison of Fig. 1 and Supplementary Fig. S2 shows that the profile of formal care use was shaped by healthcare use among younger individuals, and care-home use among older individuals.

**Figure 2.**
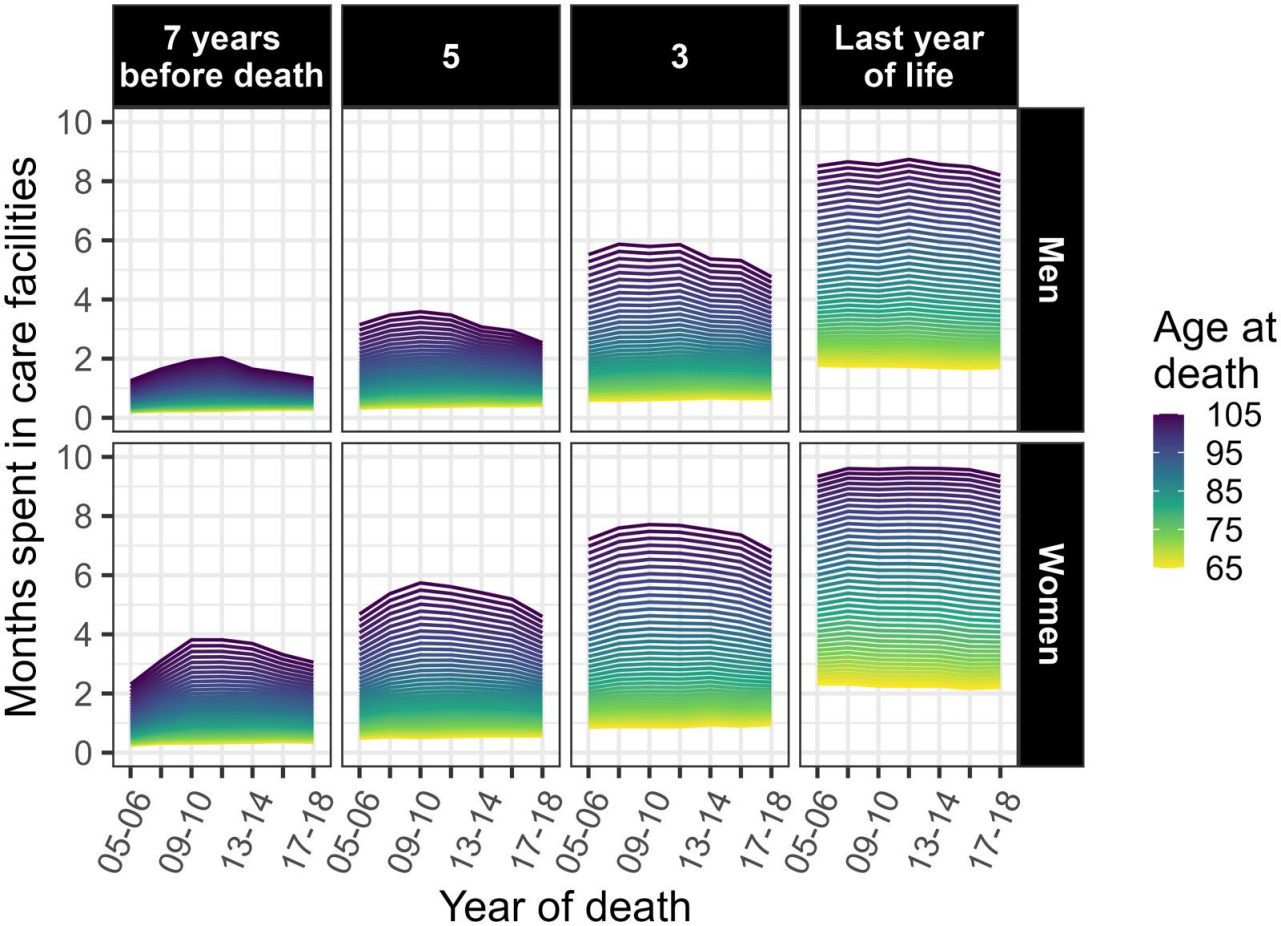
Predicted time spent in care facilities from multinomial logistic models by gender, selected time to death, year of death, and age at death. Decedents aged 65 or older in Finland between 2005 and 2018.

Figure 2 reports the predicted months spent in care from the age-year models. Younger decedents (brighter lines) spent less time than older decedents for each combination of gender, TTD, and year of death. The time spent in care increased as death approached and the differences between younger and older decedents widened, suggesting that age has a stronger effect on time in care the closer one gets to death. Throughout the study period, the largest difference in care use between men aged 65 and 105 was between one and two months seven years before death. It increased to seven months during the last year of life, when the older decedents spent 8.5 months in care. Among women, the difference of 2–3.5 months seven years before death became more than seven months in the last year of life, the oldest staying up to 10 months in care facilities. Over time, there was overall stability in the use of formal care among younger decedents, whereas older people followed an inverted U-curve that was most evident in the years further from death. More specifically, the time older men spent in care first increased and then declined, resulting in a convergence of one month among those dying at different ages. The increase in time spent in care was bigger among older women, and the decline that followed had returned to near the 2005–06 levels by 2017–18.

**Figure 3.**
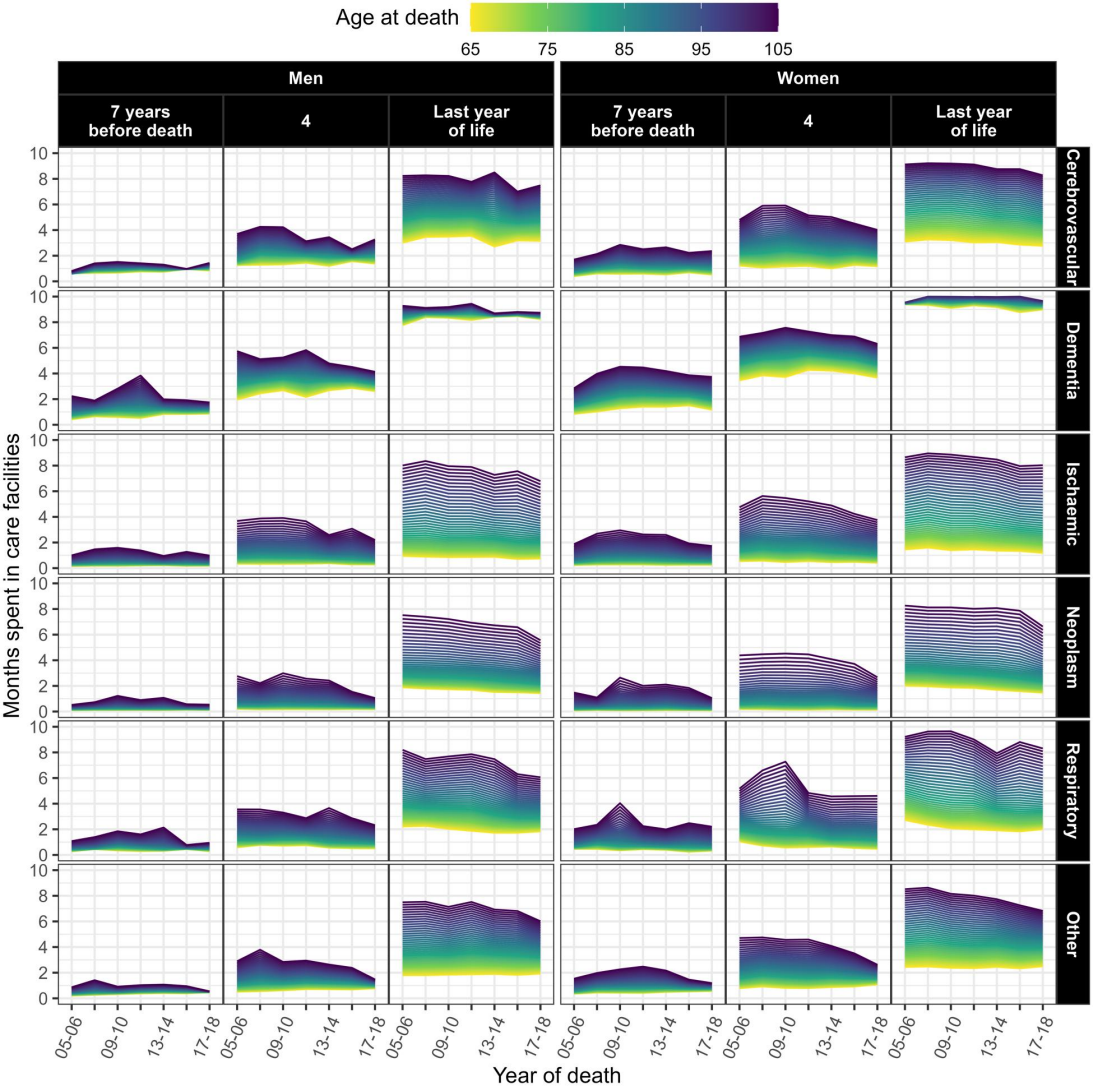
Predicted time spent in care facilities from multinomial logistic models by gender, selected time to death, year of death, age at death, and cause of death. Decedents aged 65 or older in Finland between 2005 and 2018.

As Fig. 3 shows, time spent in care varied by age and TTD for different causes of death. Individuals dying of cerebrovascular diseases and dementia spent the most time, whereas those dying of neoplasms and ischaemic diseases spent the least time. For example, men aged 85 who died of dementia in 2017–18 spent about 8.5 months in care in their last year of life, which was 6.1 months more than those who died of neoplasm. The difference was similar among women, although those dying of dementia and neoplasm were in care for 9.5 months and 3.5 months, respectively. For most causes of death there was convergence in care use between age groups over time, as shown by the brighter and darker lines getting closer. This was attributable to a reduction in use among older decedents, whereas it remained fairly stable among younger decedents. The effect of age was substantial and increased approaching death for each combination of gender and cause of death except dementia, which instead showed a decreasing effect of age approaching death, resulting in the smallest difference in care use by age in the last year of life. In fact, men aged 105 who died of dementia spent nine months of their last year of life in care, as opposed to eight months among men aged 65. Similarly, women aged 105 stayed in care for 10 months, compared with nine among those aged 65.

Supplementary Fig. S3 shows the reduction in healthcare use over time by cause of death. The effect of age appears residual compared to the effect of TTD, as the lines are more compressed around similar values. Among those who died of dementia, younger people spent more time in healthcare during their last year of life than older people, showing an inverse pattern compared to other causes of death.

Supplementary Fig. S4 shows that the use of care homes was also strongly dependent on age throughout the study period. A comparison of Supplementary Figs. S3 and S4 reveals that all causes of death, except cerebrovascular diseases and dementia, led to a decrease in healthcare usage over time, but did not increase the use of care homes. This is illustrated in Fig. 3, as time in care facilities reduced slightly for most causes of death. Given that time spent in care homes accounts for the largest proportion of total time in care, most of the conclusions we draw concerning the use of care homes are similar to those already reported for total care.

## Conclusions

Our results show that age, TTD, and cause of death were strong determinants of formal care usage in Finland between 2005 and 2018, showing few changes over time. Despite this stability in adjusted averages, there was a substantial increase in unadjusted per-capita care use and total care use on the population level. Our findings that age, TTD, and causes of death are strong predictors of care use in Finland are consistent with results reported in previous studies [15–20].

Over time, we observed an increasing concentration of care in the last years of life, showing as reduced time in care during years further from death, especially among older decedents. The decreasing importance of age over time and an increasing concentration of long-term care utilization in the last months of life between early 2000 and early 2010 have been reported for Finland [17–20]. Although long-term care does not fully overlap with our definition of care homes, our results show that this trend continued in the 2010s.

Despite the concentration of care use at the end of life and an overall stability of usage patterns, we observed a large increase on the population level. Total growth was driven mainly by the shifting of death to increasingly older ages, particularly beyond 80. Although the differences between younger and older decedents narrowed, those who died at younger ages still spent substantially fewer months in care than their older counterparts. It is well known that age is one of the main determinants of multimorbidity and cognitive impairments, and that both are tied to an increase in care use [4, 5]. In particular, usage preceding dementia-related death is extensive and begins earlier [15, 22], making dementia the main driver of the rising long-term care use [20].

We found that age mattered more in terms of time spent in care-home facilities, whereas TTD was more relevant to healthcare, as found in previous studies [11, 15, 16]. The longer time spent in care homes compared to healthcare facilities drove the utilization of formal care and how it changed over time. More specifically, we observed that age assumed slightly less importance in the use of care homes and hence of formal care in general, particularly in the years furthest from death. Over time and on average, we observed stability or reductions in care use by causes of death, age, and gender, despite the increase in both unadjusted per-capita and total use on the population level.

Given that the time spent in care homes predominates in the total time individuals spend in care, our results refute the ‘red herring’ hypothesis that age is only marginally important once TTD is considered, as Zweifel and colleagues originally suggested [9]. The influence of age in predicting the use of non-acute care has also been shown previously [11], also in the Finnish context [15, 16].

Finland witnessed two major policy shifts during the study period. First, efforts to de-institutionalize long-term care and encourage the adoption of home-like care facilities effectively phased out long-term-care services provided within health centres [28, 29]. Although the care in health centres served similar purposes as in nursing homes and service homes with 24-h assistance, health centres are administratively classified under healthcare facilities, whereas the others fall under care-home facilities. This shows in our analysis in the clear reduction in healthcare usage and a corresponding increase in the use of care-home facilities among older decedents. Second, cutting down on care-related costs, restricting eligibility criteria and encouraging ‘ageing in place’ have increasingly promoted community living as opposed to residential care through the provision of home-based services [23, 24]. The increasing concentration of care utilization at the end of life and the reduced importance of age are therefore likely to be driven by changes in policy on eligibility criteria, rather than improved population health [30]. It is also possible that these policy shifts have had implications for reliance on informal care, with older people increasingly relying on care provided by their kins (usually spouses or children), who are generally the most likely to assume major care responsibilities [31, 32]. However, further analysis is needed to evaluate the gap between the use of and the need for care, as well as the degree to which reductions in use have coincided with a greater burden on informal caregivers, or with improvements in resource optimization.

This work has limitations. First, given the lack of data on formal home-based care and informal care, we were unable to analyse how they changed over time. Although informal care was beyond the scope of this study, the time spent at home includes the unobserved use of formal home-based care. Finally, increased awareness about dementia during the study period has led to its being more frequently specified as the underlying cause of death in Finland [33] and other countries [34, 35]. This may have resulted in decedents not being diagnosed with dementia in the earlier years of observation, causing inflated reported care use related to other causes of death at the beginning of our study period.

The main strengths of this work include the large number of observations that enhanced its statistical power, as well as the high quality of the register-based data, with little loss to follow-up or self-report bias. Drawing information from Finnish registers enabled the daily-based follow-up of transitions between care facilities and home, for extensive study periods and for the entire population. Our study analysed care utilization up to seven years before death over a 14-year period, ensuring a comprehensive examination of age and TTD effects and their evolution over time by cause of death.

In sum, we observed that age and TTD are both strong determinants of formal care use. We found an increasing concentration of care usage in the last years of life for most causes of death, and a convergence among older decedents towards the levels of younger decedents. Although both trends would contribute to a reduction in usage, our study showed a striking increase in total use on the population level as well as an increase in unadjusted per-capita use, mainly due to the occurrence of death at older ages during the study period. Rising longevity will likely increase the use of care even if age-specific usage patterns are in decline or relatively constant. Governments therefore need to prepare for an increasing demand for care in the future by strengthening and supporting the whole care system so as to avoid unmet needs.

## Supplementary Material

ckaf061_Supplementary_Data

## Data Availability

The datasets analysed in the current study are not publicly available due to privacy policies.

## Use of artificial intelligence (AI) tools

Key points

- The study examined trends in the use of formal care in Finland by age, time to death, and cause of death.
- Age and time to death remained strong determinants of formal care usage for each cause of death between 2005 and 2018 in Finland.
- Over time, total care usage on the population level and unadjusted per-capita care usage increased strikingly, despite the higher concentration of use in the last years of life among older decedents.
- Rising longevity will likely increase the use of care, even if age-specific care usage declines or remains constant, posing a risk to the sustainability of the national care system.
