# Supplementary material for: Trends in formal care by age and time to death: the use of healthcare and care-home facilities in Finland between 2005 and 2018
Source: Eur J Public Health. 2025 Apr 27;35(3):434–9. doi: 10.1093/eurpub/ckaf061 (PMC12199358; doi:10.1093/eurpub/ckaf061)
Supplement: ckaf061_Supplementary_Data [file ckaf061_supplementary_data.docx]

# Trends in formal care by age and time to death: the use of healthcare and care-home facilities in Finland between 2005 and 2018 – Supplementary material

Luca Dei Bardi ^(1,2,*)^, Margherita Moretti ^(1,2)^, Laura Cacciani ^(3)^, Kaarina Korhonen ^(1,2)^, Pekka Martikainen ^(1,2,4)^

^(1)^ Helsinki Institute for Demography and Population Health, University of Helsinki, Helsinki, Finland

^(2)^ Max Planck – University of Helsinki Center for Social Inequalities in Population Health, Helsinki, Finland

^(3)^ Department of Epidemiology of the Lazio Region, ASL Roma 1, Italy

^(4)^ Max Planck Institute for Demographic Research, Rostock, Germany

^(*)^ Corresponding author, [luca.deibardi@helsinki.fi](mailto:luca.deibardi@helsinki.fi), Unioninkatu 33 00170 Helsinki Finland

**Healthcare and care home specification**

Following the grouping also used in previous studies^1,2^, healthcare refers to overnight stays and day surgery in hospitals and health centres, whereas care home includes stays in nursing homes, service housing with 24-hour assistance, and rehabilitation centres. We included day surgery to be consistent with previous literature, and to ensure a comprehensive measure of healthcare use over time. With changing treatment practices (e.g. medical procedures optimised over time), considering only overnight stays would have led to the underestimation of healthcare use in more recent years.

**Model specification**

For the statistical analyses we ran multinomial logistic Vector Generalized Linear Models (VGLM), described by Yee and colleagues^3^, and implemented in the VGAM package in R^4^. We first ran age-year models stratified by gender and time to death (TTD), each specified as:

$$\frac{{days}_{c}}{365}=age+year+age\cdot year$$

where “days” is the number of days spent in care facilities or at home (“_c_” = social care, health care, home), “age” is the age at death, “year” is the year of death specified as dichotomic variables representing couplets (2005-06, 2007-08, …, 2017-18), and “⋅” represents an interaction term between variables. Finally, we ran our complete models, also stratified by gender and TTD, specified as follows:

$$\frac{{days}_{c}}{365}=age+year+cause+age\cdot year+age\cdot cause+year\cdot cause+age\cdot year\cdot cause$$

where “cause” refers to causes of death specified as a series of dichotomous variables.

We chose to stratify our analyses by TTD to handle multiple observations for the same individuals over time, and to have independent observations within each model. We included all possible interactions in our models so that the values of each variable could vary with the values of the others and thus provide a better prediction of the outcome. As an example, the interaction between age, year and cause of death in the complete model does not assume/force the effect of age to be the same through years and causes of death, allowing it to vary.

**Supplementary figures**


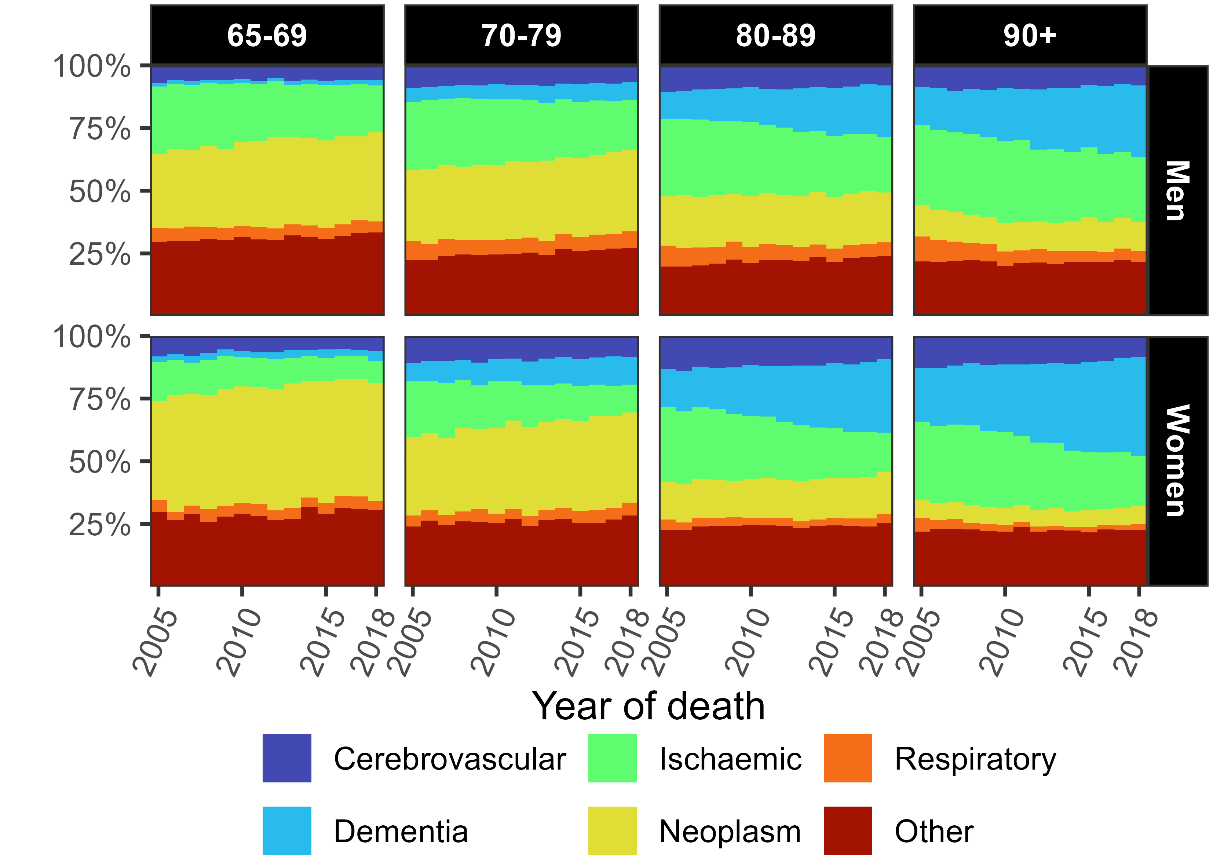


**Figure S1**: Proportions of deaths by age at death, gender, year of death and causes of death. Decedents in Finland aged 65 or older who died between 2005-2018.


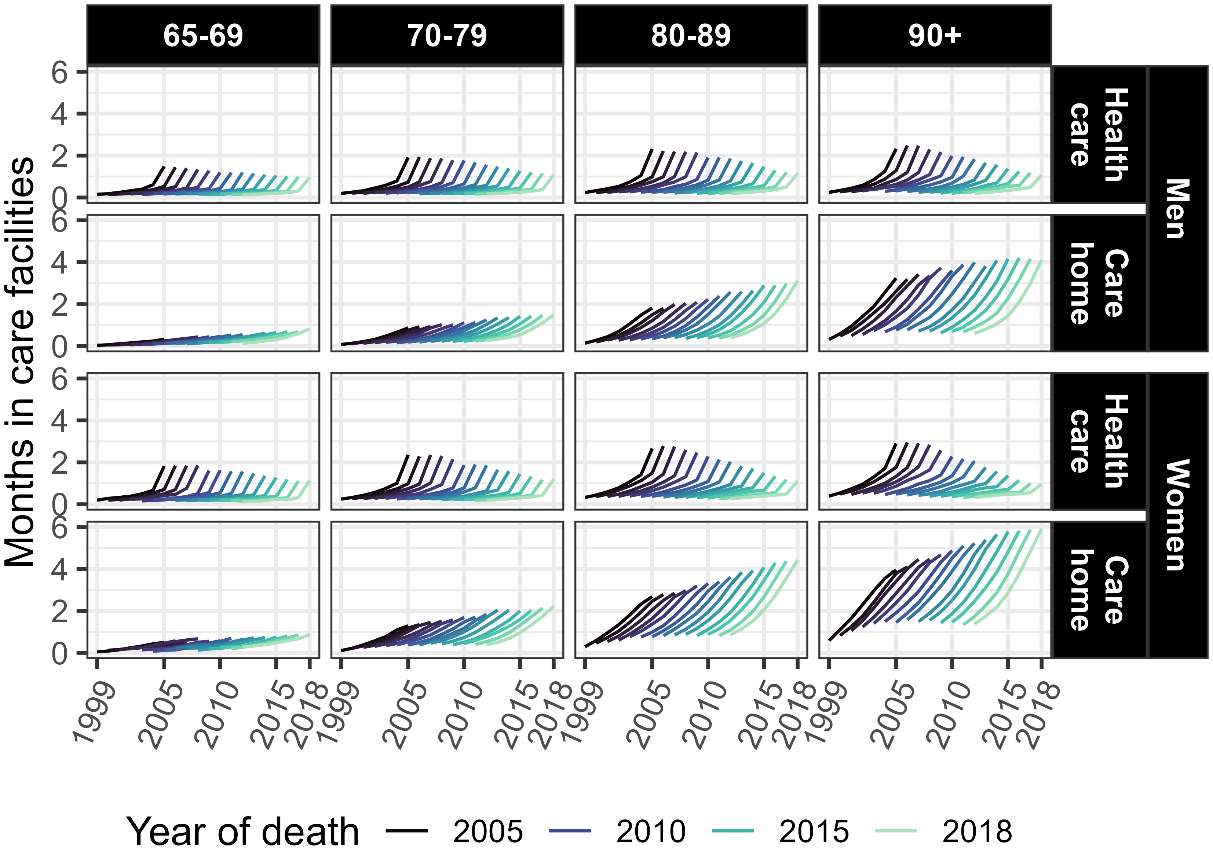


**Figure S2**: Average number of months spent in care facilities in the last seven years of life by age at death, gender, type of care, year of death and time to death. Decedents aged 65 or older in Finland between 2005-2018.


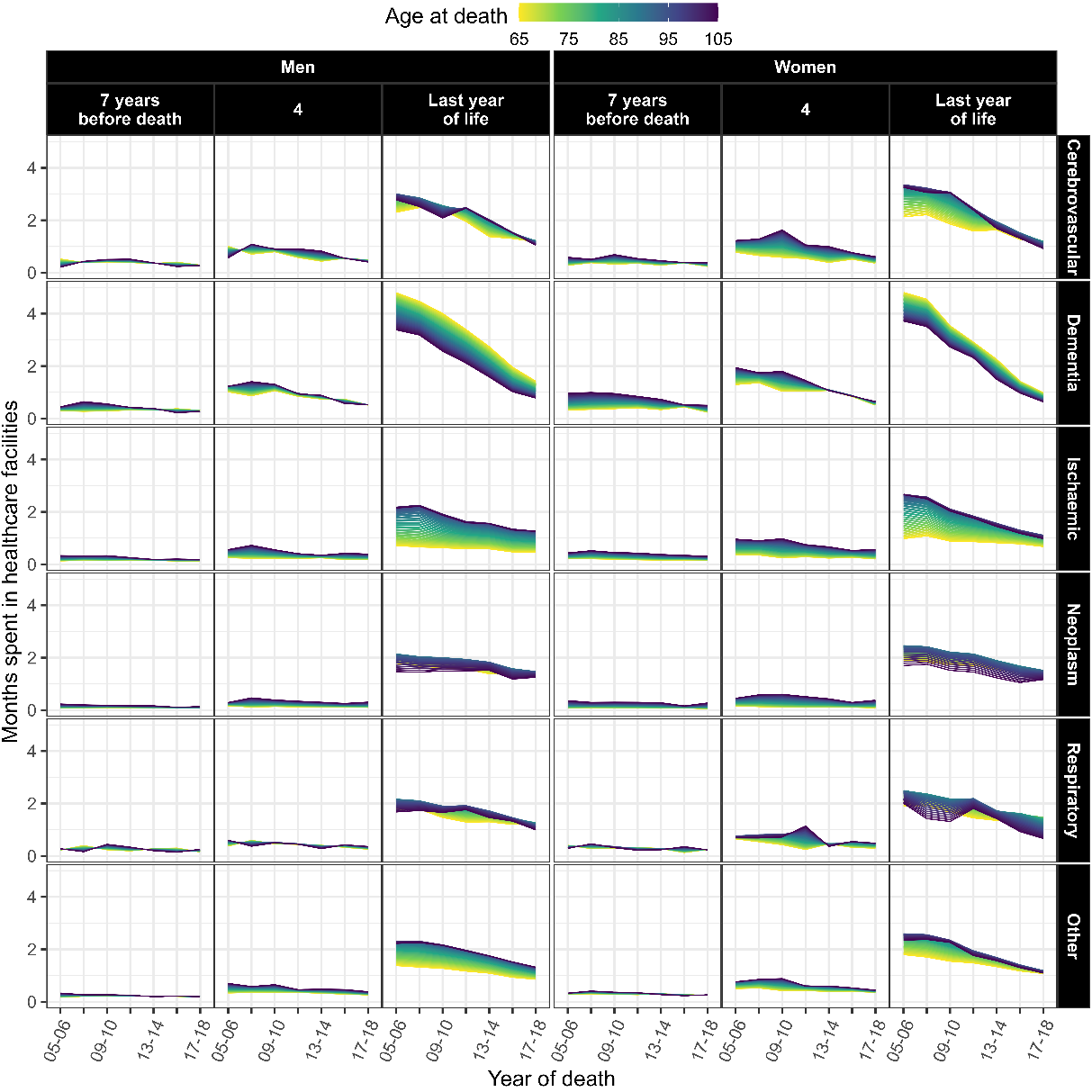


**Figure S3**: Predicted time spent in healthcare facilities from multinomial logistic models by gender, selected time to death, age at death, year of death and cause of death. Decedents aged 65 or older in Finland between 2005-2018.


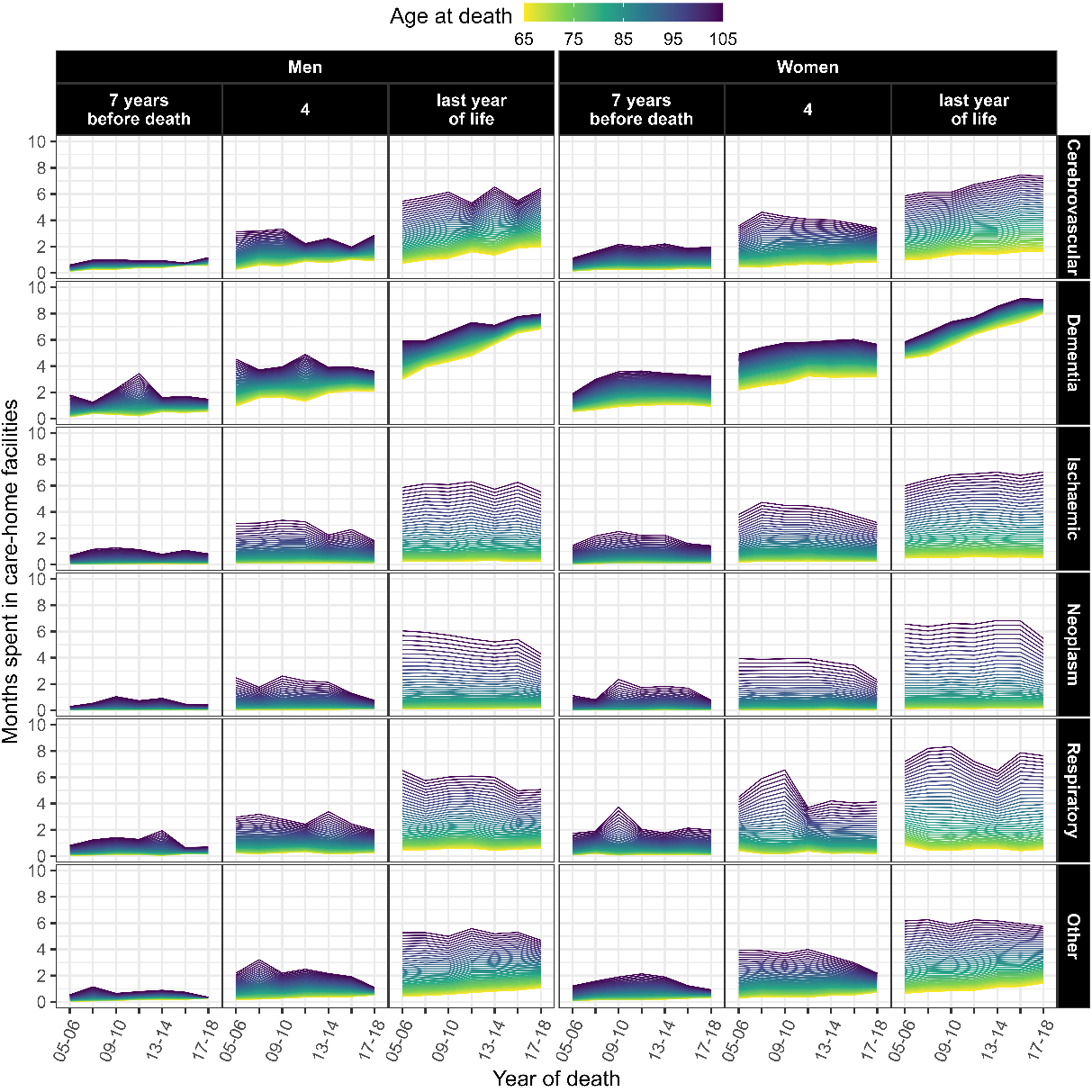


**Figure S4**: Predicted time spent in care-home facilities from multinomial logistic models by gender, selected time to death, age at death, year of death and cause of death. Decedents aged 65 or older in Finland between 2005-2018.

## References used in the supplementary material

1 Martikainen P, Murphy M, Metsä-Simola N, Häkkinen U, Moustgaard H. Seven-year hospital and nursing home care use according to age and proximity to death: variations by cause of death and socio-demographic position. *J Epidemiol Community Health* 2012;66:1152–58.

2 Murphy M, Martikainen P. Use of hospital and long-term institutional care services in relation to proximity to death among older people in Finland. *Soc Sci Med* 2013;88:39–47.

3 Yee TW, Wild CJ. Vector Generalized Additive Models. *J R Stat Soc Ser B Methodol* 1996;58:481–93.

4 Yee TW. The VGAM Package for Categorical Data Analysis. *J Stat Softw* 2010.
